# Antibiotic resistance in patients with chronic ear discharge awaiting surgery in Nepal

**DOI:** 10.5588/pha.21.0029

**Published:** 2021-11-01

**Authors:** R. R. Karn, R. Acharya, A. K. Rajbanshi, S. K. Singh, S. K. Thakur, S. K. Shah, A. K. Singh, R. Shah, S. Upadhya Kafle, M. Bhattachan, A. Abrahamyan, H. D. Shewade, R. Zachariah

**Affiliations:** 1 Nepal Netra Jyoti Sangh/Eastern Regional Eye Care – Programme/Biratnagar Eye Hospital, Biratnagar, Nepal; 2 World Health Organization, Country Office, Kathmandu, Nepal; 3 Tuberculosis Research and Prevention Center, Yerevan, Armenia; 4 International Union Against Tuberculosis and Lung Disease (The Union), Paris, France; 5 The Union, South East Asia, New Delhi, India; 6 United Nations Children’s Fund/United Nations Development Programme/World Bank/WHO Special Programme for Research and Training in Tropical Diseases (TDR), World Health Organization, Geneva, Switzerland

**Keywords:** SORT IT, operational research, surveillance, health systems, Sustainable Development Goals

## Abstract

**SETTING:**

Biratnagar Eye Hospital, Biratnagar, Nepal, which offers ear surgery for chronic suppurative otitis media (CSOM).

**OBJECTIVE:**

In patients with CSOM awaiting surgery, to determine the 1) sociodemographic characteristics 2) bacterial isolates and their antibiotic resistance patterns and 3) characteristics of those refused surgery, including antibiotic resistance.

**DESIGN:**

A cohort study using hospital data, January 2018–January 2020.

**RESULTS:**

Of 117 patients with CSOM and awaiting surgery, 64% were in the 18–35 years age group, and 79% were cross-border from India. Of 118 bacterial isolates, 80% had *Pseudomonas aeruginosa* and 16% had *Staphylococcus aureus*. All isolates showed multidrug resistance to nine of the 12 antibiotics tested. The lowest antibiotic resistance in *P. aeruginosa* was for vancomycin (29%) and moxifloxacin (36%), and for *S. aureus*, this was vancomycin (9%) and amikacin (17%). Fourteen (12%) patients underwent surgery: myringoplasty (*n* = 7, 50%), cortical mastoidectomy with tympanostomy (*n* = 4, 29%) and modified radical mastoidectomy (*n* = 3, 21%). Those infected with *P. aeruginosa* and with resistance to over six antibiotics were significantly more likely to be refused for surgery.

**CONCLUSION:**

Patients awaiting ear surgery were predominantly infected with multidrug-resistant *P. aeruginosa* and were consequently refused surgery. This study can help inform efforts for improving surgical uptake and introducing cross-border antimicrobial resistance surveillance.

## Abstract (FR)

**LIEU:**

Hôpital ophtalmologique de Biratnagar, Népal, qui propose des interventions chirurgicales de l’oreille aux patients atteints d’otite moyenne chronique suppurée (CSOM).

**OBJECTIF:**

Déterminer 1) les caractéristiques sociodémographiques, 2) les isolats bactériens et leurs profils de résistance aux antibiotiques et 3) les caractéristiques des patients se voyant refuser la chirurgie (dont résistance aux antibiotiques) chez les patients atteints de CSOM en attente de chirurgie.

**MÉTHODE:**

Étude de cohorte réalisée à l’aide de données hospitalières, janvier 2018-janvier 2020.

**RÉSULTATS:**

Sur les 117 patients atteints de CSOM en attente de chirurgie, 64% appartenaient à la tranche d’âge des 18–35 ans et 79% étaient des patients transfrontaliers en provenance d’Inde. Sur 118 isolats bactériens, 80% étaient des isolats de *Pseudomonas aeruginosa* et 16% de *Staphylococcus aureus*. Tous les isolats ont montré une résistance à neuf des 12 antibiotiques testés. Les plus faibles résistances chez *P. aeruginosa* étaient celles à la vancomycine (29%) et à la moxifloxacine (36%). Pour *S. aureus*, il s’agissait de celles à la vancomycine (9%) et à l’amikacine (17%). Quatorze (12%) patients ont subi une intervention chirurgicale : myringoplastie (*n* = 7, 50%), mastoïdectomie corticale avec tympanotomie (*n* = 4, 29%) et mastoïdectomie radicale modifiée (*n* = 3, 21%). Les patients infectés par *P. aeruginosa* avec une résistance à plus de six antibiotiques étaient significativement plus susceptibles de se voir refuser la chirurgie.

**CONCLUSION:**

Les patients en attente de chirurgie de l’oreille étaient principalement infectés par un isolat multirésistant de *P. aeruginosa,* et se sont donc vu refuser la chirurgie. Cette étude peut permettre d’orienter les efforts visant à améliorer le taux de chirurgies réalisées et à mettre en place une surveillance transfrontalière des résistances antimicrobiennes.

I am 21 years old and since childhood, I received antibiotics many times for ear infections. I have pus discharging from my ears and have difficulty in hearing. The doctor says I need ear surgery but is unable to operate because of antibiotic-resistant bacteria. (Patient X).

This patient’s story typifies the clinical history of recurrent otitis media. Otitis media is defined as the presence of inflammation in the middle ear.^1^ Chronic suppurative otitis media (CSOM) is defined as such inflammation which persists for at least 2 weeks and is accompanied by pus discharge and perforation of the tympanic membrane (eardrum).^2^ CSOM is mostly caused by bacteria and is one of the most frequent reasons for antibiotic prescriptions in children.^3^ A systematic review that included 15 of 21 WHO regions showed a global CSOM incidence rate of 4.7%; translating into 31 million cases, 22.6% (7.6 million) of which occurred annually in children under 5 years of age. An estimated 20,000 deaths occur annually from CSOM-related complications.^4^ CSOM can result in hearing loss and negatively affect school performance in children.^5^

In countries such as Nepal, CSOM is often associated with inappropriate antibiotic use.^6^,^7^ This may be due to the lack of standardised treatment guidelines, over-the-counter prescriptions, self-administration of antibiotics and limited access to laboratory facilities. In addition, high costs of second-line antibiotics may result in the repeated use of ineffective antibiotics, which may enhance resistance development.^2^,^8^ There is a well-established link between inappropriate use of antibiotics and the emergence of antimicrobial resistance (AMR).^6^,^9^

The Ear Department of the Biratnagar Eye Hospital, is a tertiary care facility located in Biratnagar, Eastern Nepal that offers care for ear diseases in people from both Nepal and India. This hospital is run by a non-governmental organisation (NGO) and treats about 79,000 patients and performs about 1500 ear surgeries annually.^9^ Surgeries include tympanoplasty, myringoplasty, ventilation tube insertion (Grommets tube) and modified radical mastoidectomy. Being a referral facility, the hospital is likely to receive chronic cases of CSOM, who have already been exposed to recurrent antibiotics.

Anecdotal information suggests that many patients who paid for and are awaiting surgery in this hospital are being denied surgery and being refunded their up-front payments. There may be different reasons why clinicians may decline for surgery, one of which may be AMR. As a first step towards quantifying this problem, the hospital management requested the monitoring and evaluation team to characterise patients, who despite being eligible for surgery, are declined for surgery. This is important to inform efforts towards reducing surgical refusals and upholding the credibility of the hospital.

There has also been a call to improve laboratory surveillance of AMR, which is an identified priority of the global and national action plans on tackling AMR.^10^,^11^ Such information is needed to track the magnitude of AMR, inform burden of disease estimates and drive local, national and cross-border actions to tackle AMR.^12^ Knowledge of the type of bacterial pathogens and their resistance patterns in CSOM may also help to inform management strategies.^13^

A PubMed search found a few studies on AMR in a tertiary referral facility and its relation to ear surgery from India, but none from Nepal, where resistance patterns might be different.^14^,^15^ Among patients presenting with CSOM and awaiting surgery in Biratnagar Eye Hospital, we determined 1) the sociodemographic characteristics of patients and if CSOM was unilateral or bilateral, 2) bacterial isolates and drug susceptibility testing (DST) patterns, 3) characteristics of those who were declined for surgery, including their DST patterns.

## METHODS

### Study design

This was a cohort study using routinely collected hospital data.

### Settings

#### General setting

Nepal is a landlocked country in South-East Asia located mainly in the Himalayas with an estimated population of 30.2 million.^16^ It borders China in the north and India in the south, east, and west. It has seven provinces with 77 districts.

#### Specific setting

Established in 2007, the Biratnagar Eye Hospital offers eye and ear care services and is managed by an NGO, the Nepal Netra Jyoti Sangh. This NGO has two ear hospitals in the country. The hospital also offers community outreach activities, and its catchment area includes Eastern Nepal and Northern India. The hospital has a well-equipped laboratory with two pathologists, one microbiologist, five laboratory assistants and two support staff.

#### Surgery for chronic suppurative otitis media

Trained paramedical staff conduct regular outreach screening in communities and schools for eye and ear conditions and refer patients to Biratnagar Eye Hospital. The hospital also receives referrals from various health care facilities, including from India. Patients arriving at the hospital are examined by a specialist ear surgeon who determines eligibility for, and the type of surgery. Once the patient consents, they are required to pay 48 Nepalese rupees (1USD = NPR113) for registration, and NPR6000–9000, depending on the type of ear surgery. Laboratory fees (NPR1500) and medications are charged extra (cost range: NPR2000–3000).

Those with ear discharge are offered empirical treatment for 1 week with amoxicillin/clavulanate and if ear discharge persists, ciprofloxacin (CPF) is offered for a further 2 weeks. The latter are asked to undergo culture testing and DST, and offered tailored antibiotic treatment. Those patients for whom the antibiotic treatment is ineffective may be referred out. Ear surgery is performed by specialised surgeons in an operating theatre and according to the existing hospital protocols.

### Sample collection, laboratory processing and antimicrobial susceptibility testing

Ear swabs were collected using aseptic techniques by trained doctors and paramedical staff and sent to the laboratory for microbiological analysis. Specimens were directly inoculated in blood agar, chocolate agar and MacConkey agar (Oxoid, Basingstoke, UK) following the standard procedures for inoculating culture media. Colonies were subcultured for purity and identified by colony morphology and biochemical tests using standard procedures. Isolates were confirmed using the API isolation system (API-Biometrieux, Basingstoke, UK). All isolated pathogens were tested for antibiotic susceptibility using the Kirby-Bauer disc diffusion technique. There are established quality control standards in place that are in line with national standards.

### Study population

The study population included all patients presenting with CSOM and undergoing bacterial culture DST at the Biratnagar Eye Hospital between January 2018 and January 2020.

### Data variables, sources of data and validation

Patient electronic records and laboratory data were used to extract relevant demographic and clinical data. Information from both data sources was merged to create one database. Each data line in the merged database was validated with information in the paper-based records. Data variables included patient identification number, age, sex, nationality, affected ear(s), bacterial culture, bacterial type and DST pattern. Variables related to surgery included surgery within 3 months of presentation and type of surgery.

### Statistical analysis

The electronic database (in MS Excel format; Microsoft, Redmond, WA, USA) was imported into EpiData software v2.2.2.186 (EpiData Association, Odense, Denmark) for analysis. Differences in characteristics of those who did and did not undergo surgery were assessed using the χ^2^ test; the level of significance was set at *P* ⩽ 0.05.

### Ethics approval

Permission to use the laboratory and electronic medical records was obtained from the programme director of the Eastern Regional Eye Care Programme. National ethics approval was obtained from the Institutional Review Committee of Biratnagar Eye Hospital, Biratnagar, Nepal (ethics approval no. BEH-IRC, 29-A, dated 29 August 2020) and the Ethics Advisory Group of the International Union against Tuberculosis and Lung Disease, Paris, France (approval No.05/20, dated 12 February 2020). As we used secondary data, the issue of informed consent did not apply.

## RESULTS

### Sociodemographic characteristics and site of chronic suppurative otitis media

Of 117 patients with CSOM who were awaiting surgery, 18% were aged <18 years, while 64% were in the 18–35 years age group, 85% had unilateral ear involvement (the rest bilateral). The majority (79%) were from India (Table 1).

**TABLE 1:** Characteristics of patients presenting with chronic suppurative otitis media and undergoing culture sensitivity testing at the Biratnagar Eye Hospital, Biratnagar, Nepal, January 2018–January 2020

Characteristics	*n*	(%)
Total	117	
Age, years		
<18	21	(18)
18–35	75	(64)
>35	21	(18)
Sex		
Male	56	(48)
Female	61	(52)
Place of residence		
India	93	(79)
Nepal	24	(21)
Ear affected		
Unilateral	99	(85)
Bilateral	18	(15)

### Bacterial isolates and DST patterns in ear discharge

Of 117 ear discharge specimens, 110 grew cultures (102 single bacteria and eight mixed bacteria (Table 2). The predominant bacteria (96% of isolates) were *Pseudomonas aeruginosa* (80%) and *Staphylococcus aureus* (16%). *P. aeruginosa* was also present in seven of the eight mixed isolates. Table 3 shows the DST patterns of 118 bacterial isolates. All isolates showed drug resistance to four or more antibiotic classes (of six classes tested), and at least nine of the 12 antibiotics.

**TABLE 2:** Bacterial types among patients presenting with chronic suppurative otitis media, Biratnagar Eye Hospital, Biratnagar, Nepal, January 2018–January 2020

	*n*	(%)
Total specimens submitted for culture	117	
Culture-positive for any bacteria	110	
Single bacteria	102	
*Pseudomonas aeruginosa*	82	(80.4)
*Staphylococcus aureus*	16	(15.7)
*Proteus* spp.	3	(2.9)
*Klebsiella pneumonia*	1	(1.0)
Mixed bacterial species	8	
*P. aeruginosa* and *S. aureus*	6	(75.0)
*P. aeruginosa* and *Proteus* spp.	1	(12.5)
*Proteus spp*. and *S. aureus*	1	(12.5)

**TABLE 3:** Bacterial isolates (n = 118) ^*^ and antibiotic resistance among patients (n = 110) presenting with chronic suppurative otitis media, Biratnagar Eye Hospital, Biratnagar, Nepal, January 2018–January 2020

Antibiotic types	Bacterial isolates and antibiotic resistance							

	*Pseudomonas aeruginosa* (*n* = 89)		*Staphylococcus aureus* (*n* = 23)		*Proteus spp*. (*n* = 5)		*Klebsiella pneumoniae* (*n* = 1)	
			
	*n*	(%)	*n*	(%)	*n*	(%)	*n*	(%)
Aminoglycosides								
Gentamicin	84	(94)	16	(70)	4	(80)	1	(100)
Amikacin	78	(88)	4	(17)	2	(40)	1	(100)
Tobramycin	76	(85)	8	(35)	1	(20)	1	(100)
Fluoroquinolones								
Ciprofloxacin	77	(87)	18	(78)	2	(40)	1	(100)
Oflaxacine	57	(64)	10	(43)	1	(20)	1	(100)
Moxifloxacin	32	(36)	6	(26)	No resistance		1	(100)
Cephalosporins								
Cefuroxime	83	(93)	11	(48)	1	(20)	1	(100)
Ceftriaxone	59	(66)	11	(48)	1	(20)	1	(100)
Ceftazidime	60	(67)	16	(70)	2	(40)	1	(100)
Chloramphenicol	66	(74)	6	(26)	3	(60)	1	(100)
Sulfonamide								
Cotrimoxazole	85	(94)	11	(48)	4	(80)	1	(100)
Glycopeptide								
Vancomycin	26	(29)	2	(9)	No resistance		1	(100)

^*^ Of the 110 culture-positive specimens, 8 had mixed growth (two organisms per specimen).

For *P. aeruginosa,* the lowest antibiotic resistance was for vancomycin (29%) and moxifloxacin (36%). For *S. aureus*, this was for vancomycin (9%) and amikacin (17%). *Klebsiella pneumoniae* (one isolate) was totally resistant to all 12 antibiotics.

### Characteristics of those denied surgery and associated risk factors.

Among 117 patients who were awaiting surgery, only 14 (12%) received surgery. The surgical interventions included myringoplasty (*n* = 7, 50%), cortical mastoidectomy with tympanostomy (*n* = 4, 29%) and modified radical mastoidectomy (*n* = 3, 21%). Table 4 compares the characteristics of those who did and did not undergo surgery. Patients infected with *P. aeruginosa* and those with resistance to over six antibiotics were significantly less likely to receive surgical care.

**TABLE 4:** Characteristics of patients with chronic suppurative otitis media who did and those who did not undergo surgery at the Biratnagar Eye Hospital, Biratnagar, Nepal, January 2018–January 2020

		Surgery				*P* value^*^

		Yes		No		
	
		*n*	(%)	*n*	(%)	
Total, *n*	110	14		96		
Age, years						
⩾18	89	11	(12)	78	(88)	0.72
<18	21	3	(14)	18	(86)	
Sex						
Female	57	8	(14)	49	(86)	0.67
Male	53	6	(11)	47	(89)	
Place of residence						
India	85	10	(12)	75	(88)	
Nepal	25	4	(16)	21	(84)	0.73
Ear affected						
Unilateral	93	9	(10)	84	(90)	
Bilateral	17	5	(29)	12	(71)	0.02
Type of bacteria						
*Pseudomonas aeruginosa*	89	6	(7)	83	(93)	0.001
Other	21	8	(38)	13	(62)	
Resistance pattern						
Resistant to ⩾6 antibiotics	94	8	(8)	86	(92)	
Resistant to <6 antibiotics	16	6	(38)	10	(62)	0.001

^*^ Pearson’s χ^2^ or Fisher’s exact test.

## DISCUSSION

This first study from a tertiary hospital in Nepal showed that the vast majority of patients with CSOM and awaiting surgery were adults, had crossed over from India and were predominantly infected with multidrug-resistant *P. aeruginosa* that was resistant to 12 commonly used antibiotics classified by the WHO as being “highly or critically important antimicrobials for use in human medicine”.^17^ These individuals were significantly less likely to receive surgical care.

The study findings are important from a local and regional perspective. On the local side, the findings point a “finger of blame” to antibacterial resistance as a reason for patients being denied ear surgery at the tertiary hospital. This knowledge can inform efforts towards tackling this challenge. From a regional perspective, the cross-border nature of patients suggests the potential for the acquisition and spread of multidrug-resistant bacteria between countries.

The study strengths are that culture and DST were performed according to international standards with quality control measures in place; the antibiotics tested included those listed by the WHO as being highly or critically important in human medicine; data were independently validated; and the study addressed an identified local and national research priority for tackling AMR. To ensure the completeness and quality of reporting, we also adhered to STROBE (Strengthening the Reporting of Observational Studies in Epidemiology) guidelines for the conduct and reporting of this observational study.^18^

The study limitations are that the bacteria were not tested for resistance to last resort antibiotics such as carbapenems (e.g., imipenem) and monobactams (e.g., aztreonam), and we are thus unaware of the level of resistance to such antibiotic classes. We also do not know the exact reasons why clinicians turned down surgery in patients with antibiotic resistance. These aspects would merit further quantitative and qualitative research. Our sample size was also not adequately powered to derive adjusted risk estimates. Finally, as the cohort included only a few children, the DST patterns in this study may not be representative for children.

The study findings have some policy and practice implications. First, 96% of CSOM ear discharges had either *P. aeruginosa* or *S. aureus*, of which respectively 87% and 78% were resistant to CPF.

The current hospital protocol for CSOM (prior to surgery) involves empirical treatment with amoxycillin/clavulanate for 1 week and if ear discharge persists, a further 2 weeks of CPF. The high levels of resistance to CPF imply that this empirical regimen is ineffective, and will add unnecessary visits and indirect costs to the health system and patient. A logical way forward would be to offer all patients at the time of presentation, culture and DST and then, tailored antibiotic therapy. Patients must also be offered counselling and made aware that background antibiotic resistance levels are high and in the absence of access to effective antibiotics, surgical care may not be possible. This is necessary to foster early patient engagement in the decision-making process and limit the loss of credibility if surgery is turned down.

Second, organisms such as *P. aeruginosa, S. aureus* and *K. pneumonia* are well known to depict high levels of resistance to commonly used first-line and second-line antibiotics. Studies from Singapore^19^ and India^20^,^21^ have yielded results similar to those in our study, where the most common bacteria implicated in CSOM were *P. aeruginosa* and *S. aureus* (in 48–98% of ear discharges) and multidrug resistance was common. *P. aeruginosa* was found to be susceptible to carbapenems (85%).^22^

There is thus a clear need to include newer-generation antibiotics as part of the armamentarium against CSOM in Nepal. This could include carbapenems and monobactams and include antibiotic testing for these. Without access to such antibiotics, it is understandable that surgeons will shy away from surgical procedures due to the risks of triggering complications, including disseminated sepsis, skull bone osteomyelitis, meningitis and even, death.^4^ Providing access to such antibiotics might positively influence surgical uptake, but the feasibility issues, such as how and who will cover the related costs, will need to be discussed at the hospital management level. However, the total number of patients translates to an average of 39 per year and should be affordable.

Third, the fact that bacteria which are known to be notorious culprits for intractable nosocomial infections were predominant is of great concern. This information should serve as a wake-up call to strengthen vigilance on infection prevention and control (IPC) measures. As the hospital also deals with eye surgery, the highest standards of IPC, along with awareness raising among health workers and patients, is paramount to prevent resistant eye infections.

Finally, the fact that close to 80% of CSOM were cross-border patients from India is an ‘eye and ear opener’ for dialogue on broadening ongoing AMR surveillance to include cross-border parameters. In a manner of speaking, ‘antibiotic resistance there is antibiotic resistance here, is antibiotic resistance everywhere’.

In conclusion, patients awaiting surgery at the Biratnagar Eye Hospital were predominantly infected with multidrug-resistant *P. aeruginosa* and these patients are being declined for surgery. Providing access to newer-generation, third-line antibiotics might be a key to improving the management of CSOM and surgical up-take in the setting.
